# Active Surface-Enhanced Raman Spectroscopy (SERS): A Novel Concept for Enhancing Signal Contrast in Complex Matrices Using External Perturbation

**DOI:** 10.1177/00037028241267898

**Published:** 2024-08-07

**Authors:** Sara Mosca, Megha Mehta, William H. Skinner, Benjamin Gardner, Francesca Palombo, Nicholas Stone, Pavel Matousek

**Affiliations:** 1 Central Laser Facility, Research Complex at Harwell, STFC Rutherford Appleton Laboratory, UKRI, Harwell Campus, Oxfordshire, UK; 2 Department of Physics and Astronomy, 3286 University of Exeter, Exeter, UK

**Keywords:** Raman spectroscopy, ultrasound, active surface-enhanced Raman spectroscopy, SERS, transmission Raman, spatially offset Raman spectroscopy, SORS, deep Raman spectroscopy

## Abstract

Noninvasive detection of surface-enhanced Raman spectroscopy (SERS) signals from deep within tissue represents a common challenge in many biological and clinical applications including disease diagnosis and therapy monitoring. Such signals are typically weak and not readily discernible from often much larger Raman and fluorescence background signals (e.g., from surrounding tissue). Consequently, suboptimal sensitivity in the detection of SERS signals is often achieved in these situations. Similar issues can arise in SERS measurements in other diffusely scattering samples and complex matrices. Here, we propose a novel concept, active SERS, for the efficient retrieval of SERS signals from deep within complex matrices such as biological tissues that mitigates these issues. It relies on applying an external perturbation to the sample to alter the SERS signal from nanoparticles (NPs) deep inside the matrix. A measurement with and without, or before and after, such perturbation then can provide powerful contrasting data enabling an effective elimination of the matrix signals to reveal more clearly the desired SERS signal without the interfering background and associated artifacts. The concept is demonstrated using ultrasound (US) as an external source of perturbation and SERS NPs inserted deep within a heterogeneous tissue phantom mimicking a cluster of NPs accumulated within a small target lesion. The overall SERS signal intensity induced by the applied US perturbation decreased by ∼21% and the SERS signal contrast was considerably improved by eliminating subtraction artifacts present in a conventional measurement performed at a neighboring spatial location in a heterogeneous tissue sample. Although the technique was demonstrated with SERS gold NPs with a standard Raman label, it is envisaged that active SERS NPs (both the nanoscale metal geometry and Raman label) could be specifically designed to deliver an augmented response to the external stimulus to further enhance the achievable SERS signal contrast and yield even greater improvement in detection sensitivity. The method was demonstrated using transmission Raman spectroscopy; however, it is also applicable to other Raman implementations including spatially offset Raman spectroscopy and conventional Raman spectroscopy performed both at depth and at surfaces of complex matrices.

## Graphical abstract

**Graphical abstract:**
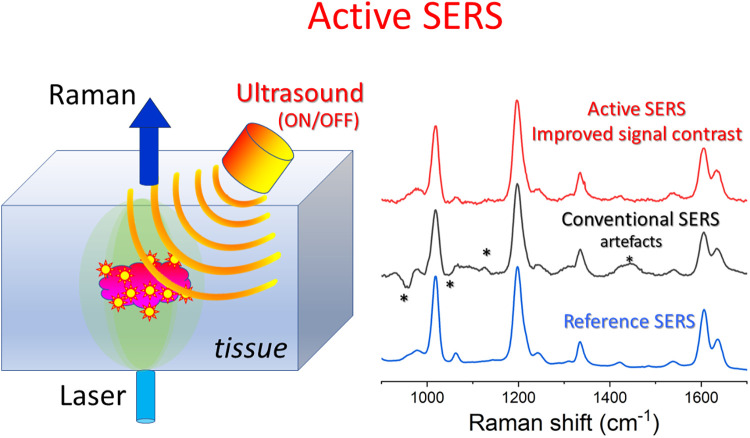
This is a visual representation of the abstract.

## Introduction

Surface-enhanced Raman spectroscopy (SERS) has gained traction in recent years, opening the prospect for applications in many analytical areas including biomedicine and disease diagnosis. However, the detection of SERS signals^1^ from target specimens at depth in the presence of intense Raman and fluorescence background signals due to the surrounding matrix is still a major challenge limiting progress in those areas. Examples of pertinent applications include the early detection and classification of cancers using functionalized SERS nanoparticles (NPs) at depth and the monitoring of disease treatments.^2–4^ In these applications, inherently weak SERS signals are often present due to low levels of target bioanalytes and/or the high depths from which the signals from the lesion can originate. Although a considerable improvement in the detectability of these signals can be achieved by combining SERS and spatially offset Raman spectroscopy (SORS)^5^ in a modality termed SESORS,^2,6^ even this approach can result in SERS signals being too weak for many potential in vivo clinical applications. As such there is a general need to boost the sensitivity of SERS signal detection in these scenarios. Increasing the concentration of SERS NPs presents a potential pathway for overcoming these issues, however, this approach is not always viable, especially with in vivo situations where often only very limited NP doses can be administered due to challenges associated with delivering NPs to the tumor site^7^ and safety considerations.

In cases where the SERS signal cannot be readily discerned as distinct Raman bands from an intense background, additional signal recovery methods are required. The most common approach is obtaining a reference (contrasting) spectrum acquired in the absence of NPs. This can be done by performing an additional measurement near the lesion to obtain a pure Raman signature of the matrix (e.g., tissue), but far enough from the NPs zone to ensure no or minimal SERS signal contribution is present. The difference between these spectra with an appropriate scaling factor (SF) will approximately remove the matrix contribution and reveal the SERS signal of the lesion.^8^ (In a limited number of scenarios where the location of the lesion is known a priori, this can also include the measurement of the probed location before NPs are introduced into the tissue.) Alternatively, a series of measurements can be performed at different locations in the vicinity and over the lesion, e.g., a linear scan, or a two-dimensional (2D) map, followed by multivariate data analysis applied to extract the desired SERS signal from the available data.^9^ These approaches, however, have often limited effectiveness due to inherent sample heterogeneity.

Crucially, the effective detection of NPs at depth is further complicated by self-absorption of Raman and other signals of the matrix (e.g., due to near-infrared (NIR) absorption) giving rise to Raman intensity attenuation that can be nonuniform across the Raman spectrum.^10^ The degree of this spectral distortion varies with the depth from which the signal originates and affects both the matrix and the SERS signal itself.^11^ In combination with commonly used linear multivariate data reduction techniques, such as principal component analysis or multivariate curve resolution, this typically leads to the generation of a large number of spurious eigenvectors upon the data reduction, as a result of the presence of nonlinear spectral distortions, yielding Raman-like spectral artifacts that can further mask real NPs SERS signals. Notably, this effect is present even when the matrix is homogenous, and its thickness or/and depth of lesion varies for the mapped points. Although this effect can be partially numerically corrected, its full elimination is not typically possible.^10^

Intense interfering fluorescence backgrounds generated by the surrounding matrix (and also potentially from ambient light) also lead to spectral artifacts^12^ that can further compromise the SERS signal detection.

**Figure 1.**
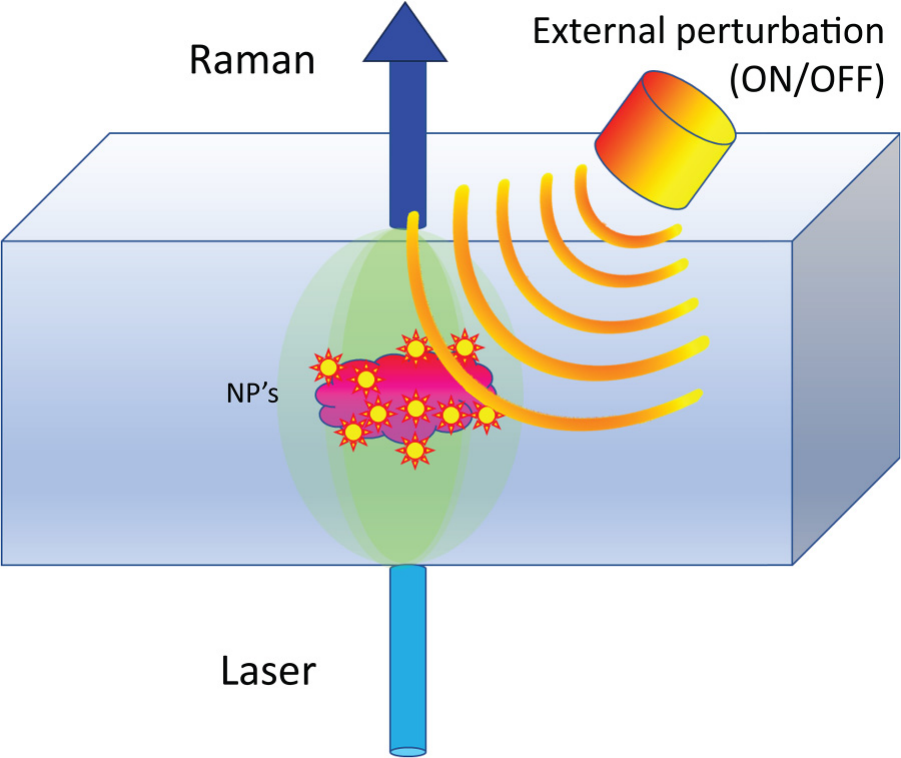
Active SERS concept: SERS signal is detected from NPs embedded in a turbid matrix in synchrony with an external perturbation that induces a SERS spectral variation.

To counteract these limitations, we propose a novel approach that enables contrast enhancement of SERS signals by suppressing the above-mentioned spectral artifacts. The technique, termed “active SERS”, relies on altering the SERS spectral intensity or its profile by an external stimulus applied transiently or in an intermittent fashion (e.g., ON/OFF) and detecting in synchrony the Raman signal from the sample (Figure 1). The Raman spectra corresponding to the two states are separately added to two spectra each corresponding to one of the states of the perturbation (e.g., ON/OFF, or before and after the perturbation). The two summed spectra are then subtracted from each other (potentially with a small SF applied to correct for any residual differences in intensities of the individual spectra) to suppress the matrix Raman spectrum and reveal the SERS spectral difference induced by the external perturbation. Alternatively, a kinetic series of spectra can be acquired and processed by multivariate analysis.

The SERS spectral change induced by the external perturbation could be in the form of a signal intensity change, as utilized here, inducing spectral shift (e.g., by NP-induced temperature change^13^) or SERS spectral profile change (e.g., new bands appearing or disappearing due to conformational changes of the SERS label). The change can either be reversible (the SERS spectrum is restored after the perturbation, with or without a delay) or nonreversible (e.g., a monotonous decay of the SERS signal).

The external perturbation could take multiple forms including electromagnetic radiation, oscillating magnetic field, ultrasound (US), or direct thermal or mechanistic stimulus. An important point is that the perturbation needs to reach the sampling zone noninvasively. In this study, we employed US, chosen principally for its ability to readily penetrate inside the most relevant matrices including biomedical tissues, and for its wide usage in clinical applications. We used a high-intensity US emitter placed in direct contact with the surface of a tissue sample. The SERS signal from an inclusion located deep inside the tissue was monitored using transmission Raman spectroscopy (TRS). The overall SERS signal intensity detected by active SERS was considerably improved by eliminating subtraction artifacts present in a conventional measurement performed at neighboring spatial locations. The proposed method can be used readily in situations where the location of NPs is known (e.g., repeated measurements of a lesion to assess its response to treatment) or to improve the purity of the spectra in the presence of large heterogeneity. It can also be used where the location of NPs is unknown (e.g., cancer screening). In this situation, the technique could be deployed by mapping a suspected zone, e.g., by moving the sample under the instrument and/or vice versa whilst searching for the SERS signal. The active SERS method is analogous to fluorescence-based enhancement methods proposed and demonstrated earlier.^14–16^

## Materials and Methods

### Silica-Coated 1,2-Bis(4-pyridyl)ethylene (BPE)-Labeled Gold Nanoraspberries (AuNRBs)

For active SERS measurements, we have used silica-encapsulated BPE-labelled gold nanoraspberries which were synthesized as described in detail in Figure S1 (Supplemental Material). Briefly, gold nanoraspberries were labeled with 1 mM 1,2-bis(4-pyridyl)ethylene (BPE) as a Raman reporter by chemisorption onto the surface and further encapsulated with silica to provide chemical and mechanical stability. Samples were characterized in solution before and after active SERS measurements to understand the behavior of AuNRBs after external perturbation (Figure S2, Supplemental Material).

### Active SERS Experimental Setup

**Figure 2.**
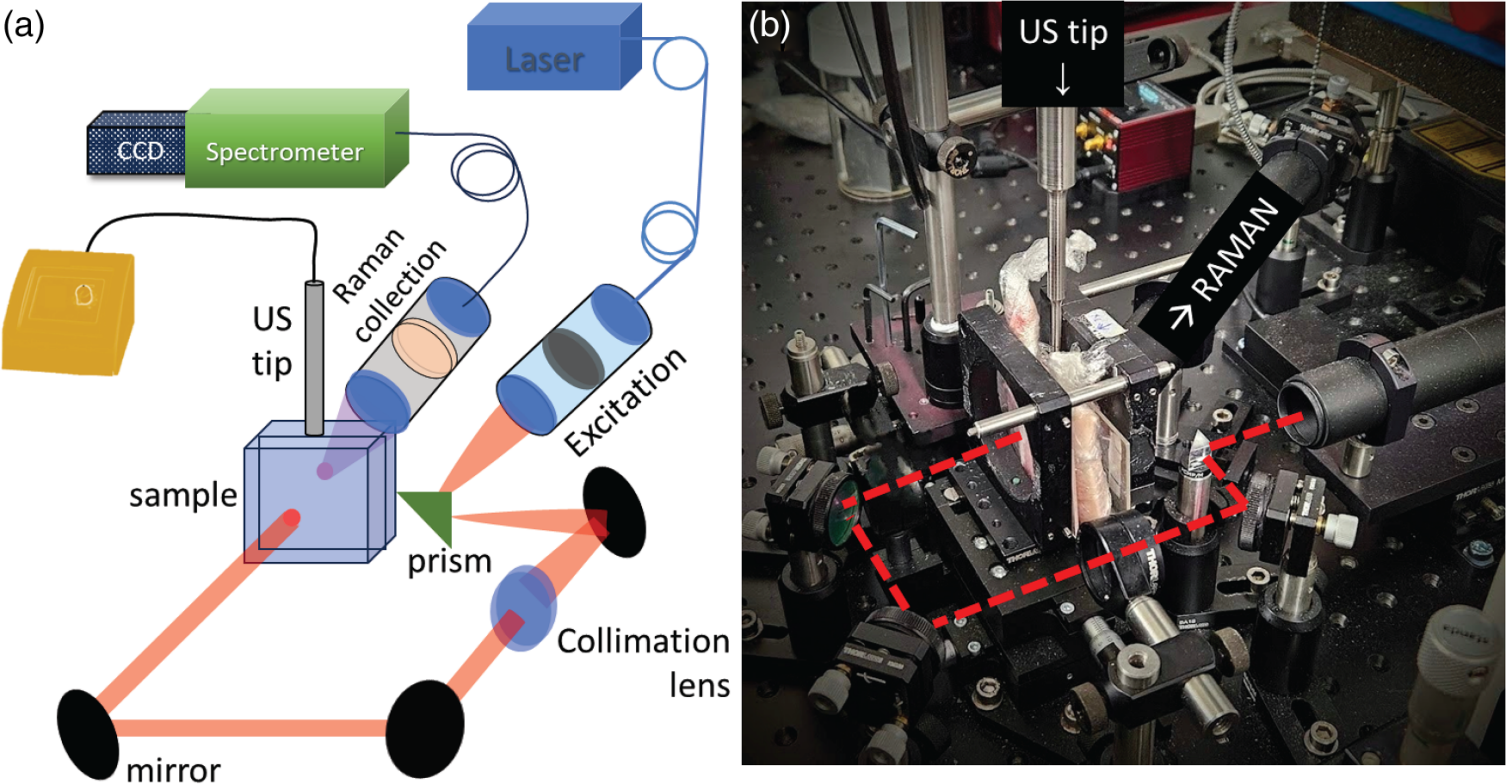
Active SERS experimental setup. (a) Schematic of the optical system, sample orientation, and US source. (b) Photograph of the set-up. The red dashed line denotes the laser beam path.

All Raman experiments were carried out using a custom-built Raman system described elsewhere^17,18^ designed to perform measurements both in transmission (TRS) and SORS geometries. In this work, we operated the system in the TRS modality. A schematic of the setup is shown in Figure 2. Briefly, we used an NIR laser (I0830MM, IPS) with 830 nm wavelength and 200 mW power (measured at the sample surface). The laser light was delivered to the sample through an optical system comprising a prism (PS910, Thorlabs) and three dielectric mirrors (BB1–E03, Thorlabs) resulting in a spot size of ∼1.5 mm on a sample surface. The Raman spectra were collected from the opposite side of the sample through a calcium fluoride window (50 × 75 × 1.5 mm, Raman grade) from an area of ∼1.5 mm diameter.

**Figure 3.**
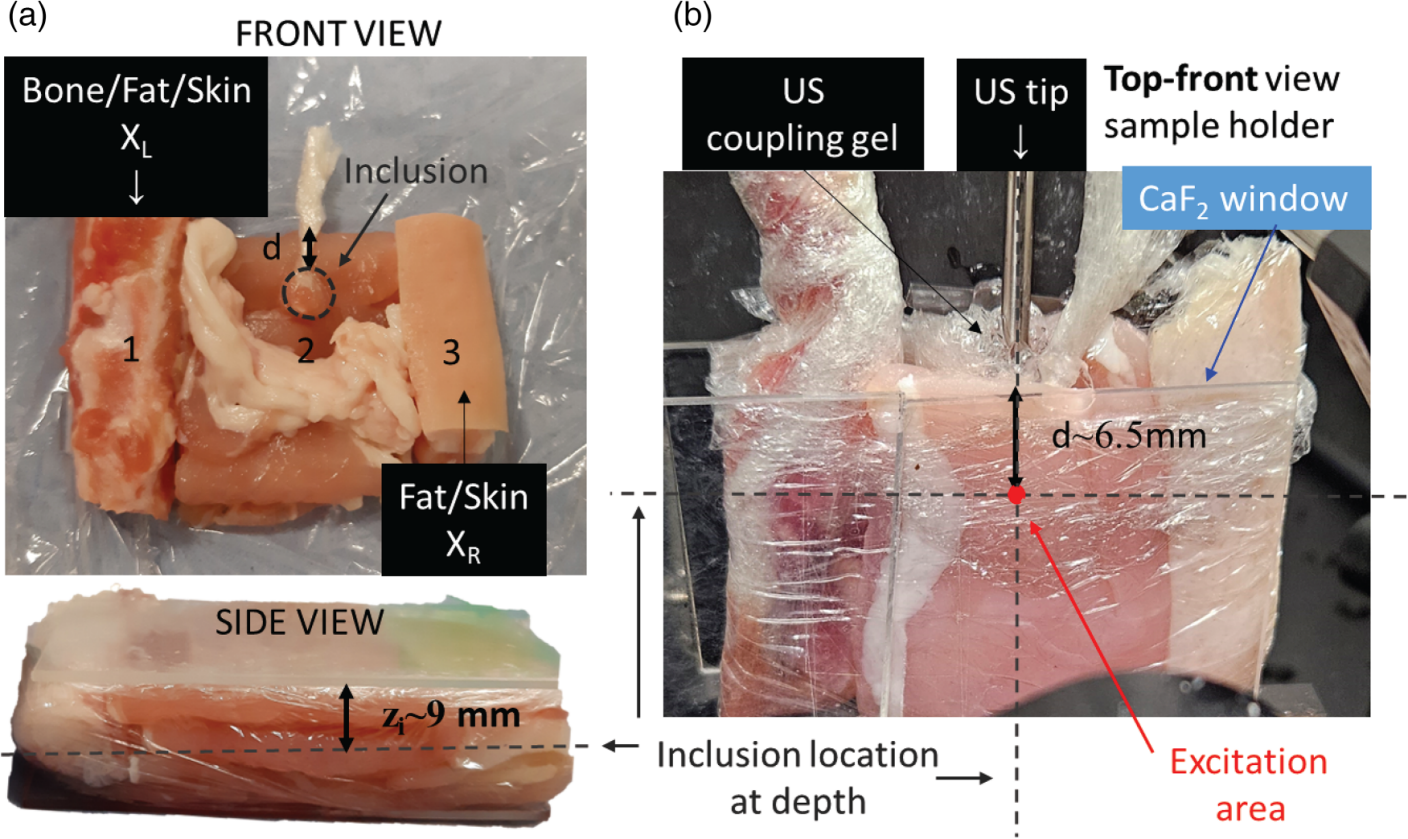
Photos of the tissue assembly (a) with all of its components (front view and side view) and (b) when located in the sample area. Copyright 2024 ACS Publications.^19^

A US sonic dismembrator (Model 50 sonic, Fisherbrand) with a 3 mm diameter tip probe was used as a US source in the active SERS experiments. The US tip was coupled to the top surface of the sample at a 90° angle to the Raman illumination–collection axis using a US coupling gel (Figures 2a and 3b). The US module was operating at a frequency of 20 kHz capable of reaching a maximum power of 50 W. The power used in the experiments reported here was 20% of the maximum output, i.e., 10 W, unless stated otherwise.

### Tissue Samples and Their Characterization

The sample consisted of an assembly of different ex vivo porcine tissues in order to mimic heterogeneous tissue in a typical biological matrix. Figure 3a shows a picture of the sample assembly with all different components: from the left to the right: (i) rib bone including flesh, fat, and skin, i.e., labeled as X_L_; (ii) a stack of bacon layers (each layer of 3 mm thickness) with a string of fat cut from the bacon rack; (iii) a skin and fat layer from a pork chop, i.e., labeled as X_R_. The overall dimensions of the assembly were 50 mm × 40 mm × 18 mm. A spherical inclusion of 5 mm diameter mimicking a tumor lesion was created using minced pork meat (5% fat) mixed with 20 μL solution of silica-encapsulated gold nanoraspberries (AuNRBs = 227 nM concentration) described above,^19^ a with BPE Raman reporter (see Section S1.1, Supplemental Material). The inclusion was embedded in the middle part of the sample (zone 2) at a depth of ∼9 mm from both the laser illumination and Raman collection surfaces (z_i_ in Figure 3a) and a distance *d *= 6.5 mm from the center of the inclusion to the top surface of the whole assembly, i.e., distance from the US tip. The ex vivo tissue assembly was wrapped in cling film and held in a sample holder compartment using two calcium fluoride (CaF_2_) windows preventing undesirable movements during the active SERS measurements. The US tip (see Figure 3b) was coupled to the top surface of the sample using a US gel, right above the inclusion.

### Measurement Settings and Data Analysis

Each active SERS measurement consisted of a kinetic acquisition of 180 Raman spectra (1 s per acquisition, five acquisitions, giving a total acquisition time of 900 s) from the central part of the ex vivo phantom in transmission Raman configuration. During the TRS kinetic acquisition, the external perturbation (US tip) was turned ON and OFF for three cycles, and overall, ON time of 375 s (T.US1 = 150 s/T.US1 + US2 = 300 s/T.US1 + US2 + US3 = 375 s). Cosmic ray removal was performed using an automatic routine (written in Matlab). Residual cosmic rays were manually removed using a median filtering (adjacent-average, around three pixels) over the different accumulation for the specific Raman wavenumber position showing the cosmic ray. No baseline subtraction postprocessing routine was applied to the active SERS data set.

Transmission Raman spectra were measured at different locations of the ex vivo sample (i.e., 20 mm to the left and to the right from the central position) to compare with the active SERS approach. (These spectra are the results of 10 averaged Raman spectra collected with 1 s, five accumulations.) A standard polynomial (order three) baseline subtraction postprocessing step was applied to the conventional TRS spectra for fluorescence removal.

The intensities of a number of dominant Raman bands, e.g., BPE main band at 1198 cm^−1^ and fat at 1451 cm^−1^, were evaluated as the areas under Gaussian curves derived from a curve fit analysis.

## Results and Discussion

In the first set of active SERS measurements, we demonstrated the basic effect of intensity changes of SERS signal upon the application of US and characterized active SERS performance. This measurement was performed at the center of the tissue phantom with the illumination and collection axis of the TRS system aligned with the center of the lesion labeled with SERS NPs as described in the Experimental section (Figure 3b).

**Figure 4.**
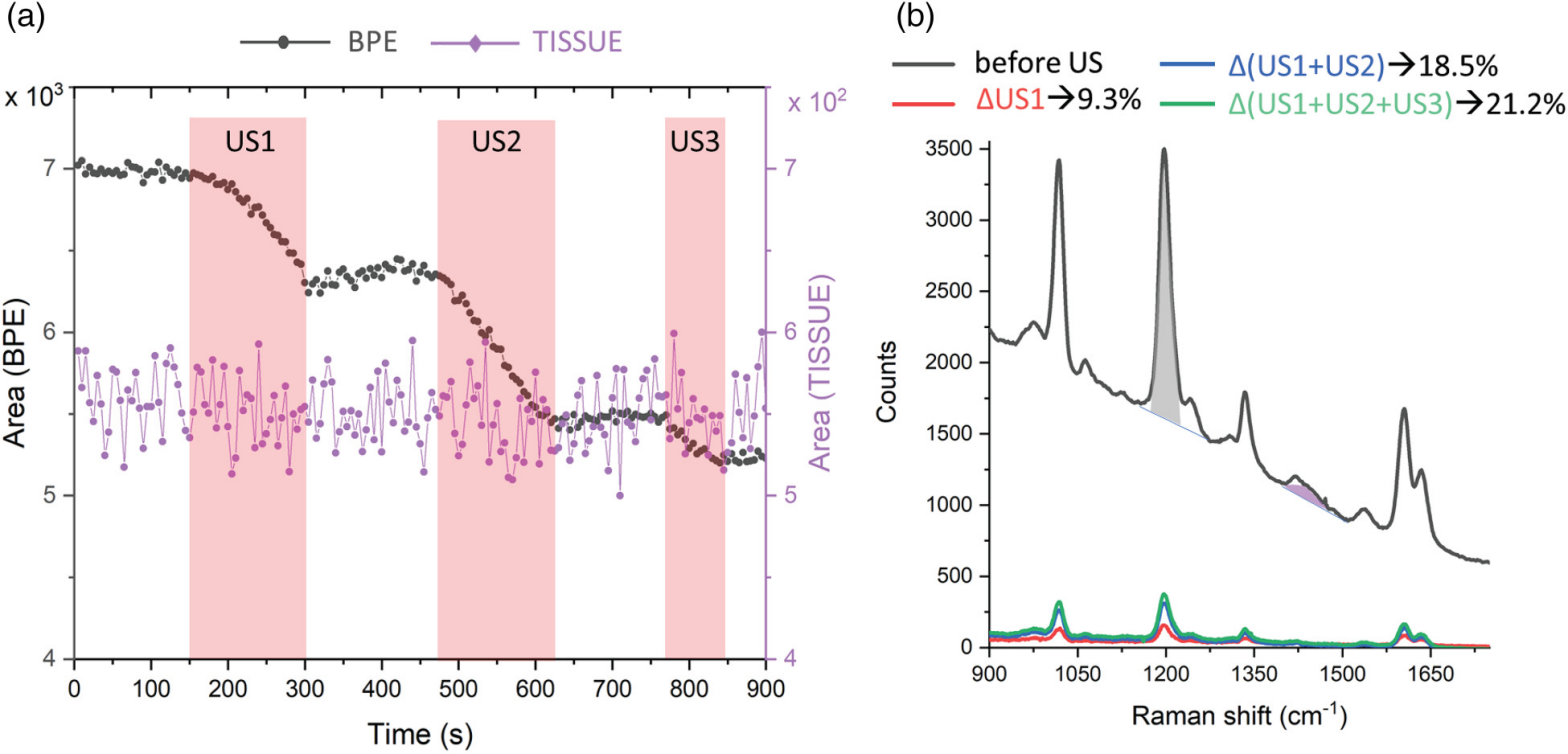
(a) Raman intensity response to US perturbation. (b) Transmission Raman spectra in the center of the ex vivo phantom before the US input (grey line). Grey and violet shaded areas highlight the BPE (1198 cm^−1^, i.e., I_0_) and fat (1451 cm^−1^) Raman bands used in the analysis. Difference Raman spectra before and after US input at different overall times of the US input are reported together with the intensity percentage drop after each US time (Δ/I_0_ × 100): T US1 (2 min 30 s, 9.3%) red line, T US1 + US2 (5 min, 18.5%) blue line, T US1 + US2 + US3 (6 min 15 s, 21%) green line.

A TRS kinetic series was acquired with the US being applied in three successive periods, whilst continuously acquiring the Raman spectra (Figure 4a). This allowed the characterization of the induced active SERS effect, the response of the system to successive applications of US, and the behavior in resting periods with no US applied. To extract this information, we plotted graphs of the evolution of the intensities of a dominant Raman band of BPE (1198 cm^−1^) and a Raman band from the tissue matrix (originating from fat at 1451 cm^−1^; Figure 4). The time periods during which the US field was switched ON are highlighted in pink. The kinetic evolution of the BPE signal shows a clear correlation with the US waves at ON and OFF states. During the ON time, the intensity of the BPE signal undergoes a considerable decrease. Upon switching OFF the US field, the SERS signal is partially restored. Comparing the SERS signal before and after the entire run indicates an induction of an overall SERS signal intensity decrease by ∼21% (see Figure 4b, Δ(US1 + US2 + US3)). In contrast, during the entire acquisition period, the Raman signal originating from the matrix did not exhibit any marked changes in intensity indicating that the US field is principally affecting the SERS NPs signal and not the Raman signal of the matrix.

The decrease in SERS intensity during US exposure is likely due to the disruption of interparticle coupling between NPs. Hot spots between NPs are known to dominate the SERS signal because of the high electric fields formed in interparticle gaps, a decreased number of hotspots would decrease the overall signal. The ultraviolet visible (UV–Vis) absorption spectra collected from NP suspensions before and after the US confirmed the loss of interparticle coupling as described in Figure S2.2 (Supplemental Material).^20,21^ It seems reasonable to conclude that some encapsulated entities include more than one NP and that these form a cluster that brings about the secondary UV–Vis absorption spectrum peak.^21^ Such clusters could then be disturbed by US waves even when embedded in a silica shell. Another explanation is that some encapsulated (or residual-free) NPs form nanoclusters which the US then disrupts. Interestingly, a UV–Vis measurement performed 30 days after the active SERS experiment revealed a partial recovery of the secondary plasmon resonance band. An additional measurement performed after 60 days did not show any substantial further recovery of this cluster band.

**Figure 5.**
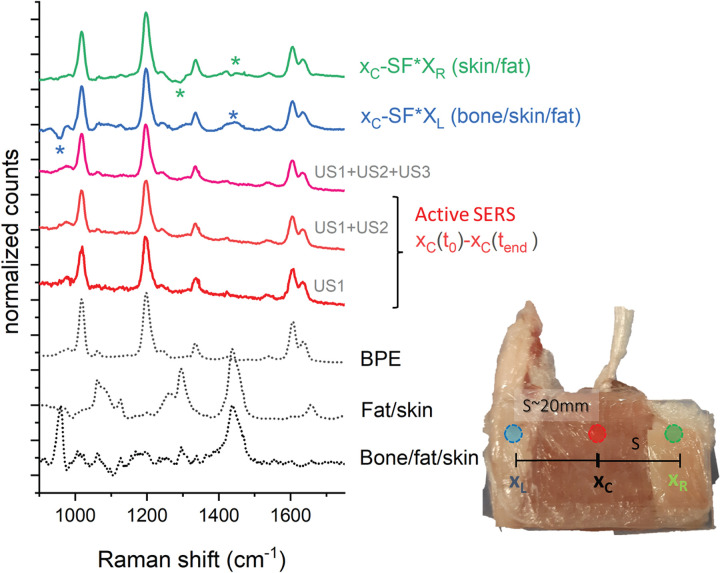
Normalized difference spectra from active SERS and lateral differences with the respective reference spectra on the bottom (black dashed lines). Spectra are vertically shifted for clarity. For the lateral difference spectra (green and blue line from the top), an SF = 0.25 was used. The same acquisition time was used for all the locations and time differences (i.e., 10 acquisitions of 1 s × 5 accumulations). An SF of 1 was used to recover the spectra from the active SERS measurements, i.e., no correcting SF was needed.

As the matrix Raman signal does not exhibit any intensity change during this kinetic series, one can subtract the TRS spectra obtained before and after the entire sequence to cancel out the matrix spectral features without incurring background artifacts and hence reveal more clearly the SERS signal originating from the lesion located within the tissue matrix. The results of this subtraction are shown in Figure 5 with evidence of the presence of BPE SERS signals uncluttered by matrix Raman spectra and residual subtraction artifacts.

**Figure 6.**
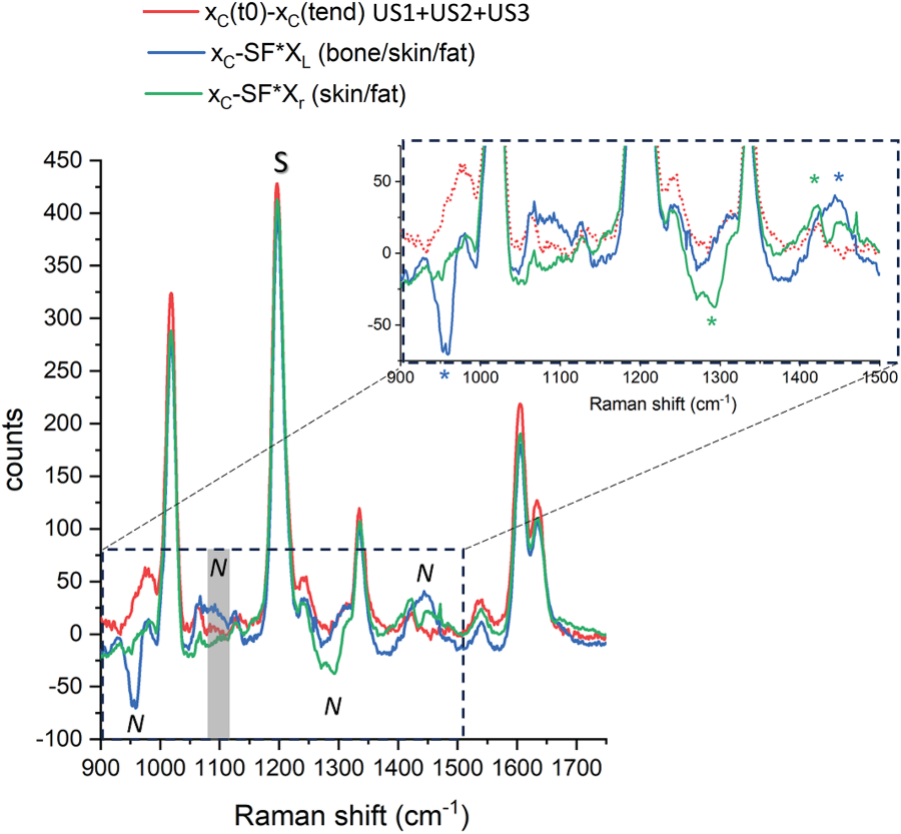
Difference spectra in absolute intensity counts – conventional Raman approach (X_C_ – SF*X_L_ and X_C_ – SF*X_R_: SF = 0.25) and the active SERS approach (X_C_(t_0_)  – X_C_(t_end_) at the end of the US input, length defined in the experimental sessions). The inset is a close-up view of the spectral artifacts due to heterogeneities (marked with an asterisk). S and N denote the Raman bands and photon shot noise and subtraction artifact regions used to evaluate the signal contrast of each approach.

This outcome is contrasted with the results of “conventional” TRS measurements, i.e., without US, obtained by subtracting “contrasting” spectra measured either at the left or the right parts of the sample from those obtained at the center of the heterogeneous sample without using US waves. These measurements were carried out before the US sequence. The section designated as the “left” part contained extra bone tissue, whilst the side designated as the “right” part contained more fat tissue and no bone. These zones were ∼2 cm away from the central part ensuring the left and right TRS probed zones were minimally overlapped with the SERS inclusion.^22^ Due to the presence of a large-scale heterogeneity of the sample, purposely introduced to emphasize the benefits of active SERS, the subtracted spectrum (with optimized scaling factors to minimize residual positive and negative peaks, SF = 0.25) is not that of pure BPE only, but is also contaminated with residual Raman signals of soft tissue, fat, and bone (Figure 6 and Table I).

**Table I.** Signal contrast evaluation for active SERS and conventional Raman methods.

	Active SERS X_C_(t_0_) – X_C_(t_end_)			Conventional method	
	US1 2 min 30 s	US1 + US2 5 min	US1 + US2 + US3 6 min 15 s	X_C_ – SF*X_L_ (bone/skin/fat)	XC – SF*XR (skin/fat)
Signal contrast	50	91	98	7	13

We defined the SERS signal contrast as the ratio of the BPE Raman band amplitude (at 1189 cm^−1^) over the intensity of the largest spurious spectral feature in the subtracted spectrum (the larger of the photon shot noise amplitude and the amplitude of the largest residual spectral band of the matrix). Specifically, for the active SERS the largest spurious spectra feature consisted of the photon shot noise amplitude around the main BPE band (calculated as standard deviation in the spectral range between 1100 and 1150 cm^−1^) while for the conventional method, this was the Raman amplitude of bone (at 960 cm^−1^) and fat (at 1440 cm^−1^), respectively for the x_L_ and x_R_ probing positions (see asterisk in Figure 6). This parameter exhibits a sevenfold improvement for the fat and a 14-fold improvement for the bone segments using the active SERS approach over the conventional method (i.e., calculated as the ratio of the signal contrast “active SERS(US1 + US2 + US3)/conventional method”; Table I). It is worth noting that by increasing the duration of US activation (i.e., from U1 to US1 + US2 and to US1 + US2 + US3) the active SERS approach led to gradually improving the quality of the recovered SERS signal (Figure 4b). However, there is, naturally, a trade-off between the quality of the recovered signal versus the overall measurement time. We wish to stress that we merely aim to highlight the qualitative effect of the suppression of any extra bands of the tissue matrix in active SERS rather than any specific quantitative improvement, as the magnitude of the improvement is dependent on the degree of heterogeneity of tissue matrix that is expected to vary considerably from sample to sample.

In a similar way, but not tested in this study, one would expect any differential self-absorption Raman artifacts discussed in the introduction to also be absent in the resulting active SERS spectrum after subtraction; as both the active SERS spectra, with and without US applied, are probing exactly the same thickness and depth of material. This should result in near complete subtraction of the matrix signals irrespective of what degree of spectral distortion from self-absorption they are subject to. Similarly, any background matrix subtraction artifacts such as charge-coupled device etaloning or detection filter ripples^23,24^ would also be expected to be removed from active SERS, as the spectra belonging to the ON and OFF states of the perturbation that are subtracted from each other would be expected to exhibit the same fluorescence, background distortion, and matrix artifacts.

This study represents only the first basic demonstration of the active SERS concept that relies on the reduction of the SERS signal upon US stimulus. It is worth mentioning that further sequential US applications, which lead to a higher signal contrast (Table I), could at the same time diminish the signal considerably and ultimately below the detection limit. This aspect represents a practical limitation of the technique. However, we envisage further major improvements to the technique to be accrued through the optimization of the SERS signals from both NPs and Raman labels for enhanced response to the external perturbation, in order to yield an even higher degree of SERS change upon the application of external stimulus and ideally also with full signal recovery after US stimulation. For example, further candidates for the active SERS method will be investigated based on a spectral shift rather than intensity decrease (e.g., due to transient conformational change induced by US, of hydrogen bond perturbation by US). This would be expected to deliver a higher contrast and a lower limit of detection of the recovered SERS signals.

## Conclusion

We described a new method, active SERS, capable of increasing the contrast of signals of interest (SERS labeled gold nanoraspberries) within the tissue by suppressing the contamination of the detected signal by matrix signals and associated artifacts. We believe the technique has a transformative potential which could be further augmented with specifically designed NPs and Raman labels exhibiting an enhanced response to external perturbation—this will be the subject of our further research.

Although the demonstration was performed with TRS, the applicability of the technique is much wider as any SERS or conventional Raman labels exhibiting spectral change upon external stimulus could be utilized to the same effect.

## Supplemental Material

sj-docx-1-asp-10.1177_00037028241267898 - Supplemental material for Active Surface-Enhanced Raman Spectroscopy (SERS): A Novel Concept for Enhancing Signal Contrast in Complex Matrices Using External Perturbation Supplemental material, sj-docx-1-asp-10.1177_00037028241267898 for Active Surface-Enhanced Raman Spectroscopy (SERS): A Novel Concept for Enhancing Signal Contrast in Complex Matrices Using External Perturbation by Sara Mosca, Megha Mehta, William H. Skinner, Benjamin Gardner, Francesca Palombo, Nicholas Stone and Pavel Matousek in Applied Spectroscopy
