# Supplementary material for: Active Surface-Enhanced Raman Spectroscopy (SERS): A Novel Concept for Enhancing Signal Contrast in Complex Matrices Using External Perturbation
Source: Appl Spectrosc. 2024 Aug 7;79(2):320–7. doi: 10.1177/00037028241267898 (PMC11823272; doi:10.1177/00037028241267898)
Supplement: sj-docx-1-asp-10.1177_00037028241267898 - Supplemental material for Active Surface-Enhanced Raman Spectroscopy (SERS): A Novel Concept for Enhancing Signal Contrast in Complex Matrices Using External Perturbation [file sj-docx-1-asp-10.1177_00037028241267898.docx]

**SUPPLEMENTARY MATERIAL**

**Active SERS: A Novel Concept for Enhancing Signal Contrast in Complex Matrices using External Perturbation**

Sara Mosca^1^, Megha Mehta^2^, William H. Skinner^2^, Benjamin Gardner^2^, Francesca Palombo^2^, Nicholas Stone^*2^, Pavel Matousek^*1,2^

1. Central Laser Facility, Research Complex at Harwell, STFC Rutherford Appleton Laboratory, UKRI, Harwell Campus, Oxfordshire, OX11 0QX, United Kingdom

2. Department of Physics and Astronomy, University of Exeter, Exeter EX4 4QL, United Kingdom

* Corresponding authors: [N.Stone@exeter.ac.uk](mailto:N.Stone@exeter.ac.uk); [Pavel.Matousek@stfc.ac.uk](mailto:Pavel.Matousek@stfc.ac.uk)

**Table of contents:**

**S1:** Synthesis of silica-encapsulated BPE-labelled gold nanoraspberries

**S1.1** Synthesis of gold nanoraspberries 2

**S1.2** Silica encapsulation and BPE labelling 2

**S.2.** Characterisation of colloidal gold nanoraspberries 3

**S2.2** SEM and TEM analysis 3

**S2.2** UV-visible spectrophotometry 4

**S3**: US power dependence on tissue assembly 5

**S4**: Longer term SERS intensity recovery after US 7

References 8

**S1: Synthesis of silica-encapsulated BPE-labelled gold nanoraspberries**

**S1.1. Synthesis of gold nanoraspberries**

For the synthesis of gold nanoraspberries (AuNRBs), a 20 mM HEPES solution was prepared by adding 2 mL of 100 mM HEPES to 8mL of ultra-pure water^1,2^. The pH of the solution was adjusted to 7.4 by adding 1M NaOH. Then, 0.25 mL of 20 mM HAuCl_4_ was added, and the colourless solution turned turbid blue within 30 to 45 minutes. The solution was left overnight at room temperature and the next day it was centrifuged at 5500 rpm for 15 minutes. The pellet was then resuspended in ultra-pure water. The size of AuNRBs was 58 nm ± 5 nm. The final concentration of gold was $4.8\times{10}^{-4}M$.

**S.1.2. Silica encapsulation and BPE labelling**

1.2ml of the AuNRBs solution was centrifuged (15 min, 4000 rpm) and redispersed in 25 µL polyvinylpyrrolidone (PVP) solution (250 mg PVP K30 in 4ml ethanol) and 450 µL ethanol. The suspension was then vortexed and further sonicated for two minutes for the complete dissolution. To this solution, 200 µL of a 1 mM (BPE) solution was added and shaken at room temperature for an hour. After incubation at room temperature overnight, the AuNRBs were centrifuged, washed with ethanol (500 µL), and redispersed in the desired dispersion medium for the addition of 3-amino-n-propyltrimethoxysilane (APTMS). 2.5 µL of 0.1% APTMS in ethanol solution was added. After incubation for 15 minutes, the dispersion was centrifuged, the supernatant was removed, and the labelled AuNRBs were redispersed in a mixture of 2-propanol (200 µL), water (80 µL), and ammonium hydroxide (2.5 µL, 28%). The solution was vortexed, sonicated for two minutes and further encapsulated by adding 11 µL of 1% TEOS in 2-propanol. The mixture was vortexed and left overnight to complete the reaction. The silica-encapsulated BPE-labelled AuNRBs were centrifuged, washed with ethanol, and redispersed in ultra-pure water for further characterisation. The final concentration of AuNRBs was 9.1 nM. For Active SERS measurements the AuNRB solution was concentrate x25 times – final concentration 227.5 nM.

**S2. Characterisation of colloidal gold nanoraspberries**

**S2.1 SEM and TEM analysis**

The shape and size of the gold nanoraspberries were determined by transmission electron microscopy (TEM) with a TEM-JEOL 2100 instrument at an operating voltage of 200 kV (model JEOL JEM 1400, Japan). Scanning electron microscopy (SEM) imaging was carried out using SEM- xT Nova Nanolab 600 FIB, a dual beam unit from FEI Quanta 250 FEG scanning electron microscope from Oxford Instruments, with a voltage of 30 kV. The sizes were determined using ImageJ software by measuring ≈50−70 individual assemblies per sample.

**
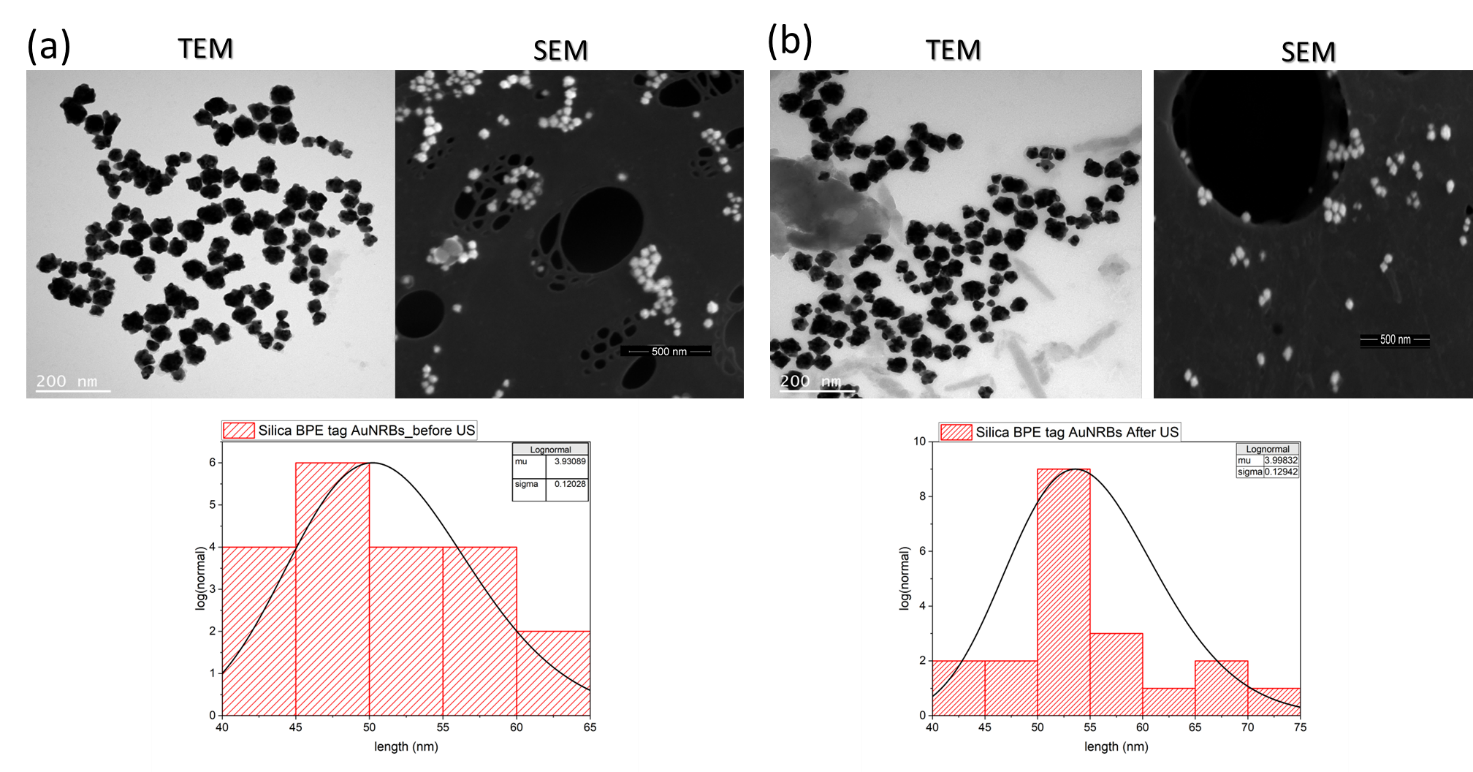
 FIG. S2.1:** TEM and SEM images and dimension population of the silica-encapsulated BPE-labelled gold nanoraspberries (a) before and (b) after the US external input (Active SERS experiment).

**S2.2 UV-visible spectrophotometry**

The UV-visible absorption spectra of the nanostructure solutions were acquired using a benchtop UV-visible spectrophotometer (Shimadzu UV-1800 UV/Visible Scanning Spectrophotometer). Absorbance measurements were performed from 370 to 970 nm with a spectral resolution of 0.2 nm inside a 5 mm pathlength quartz cuvette. Measurements were performed with the same settings before and after the US input. The experiments were performed in aqueous solution.


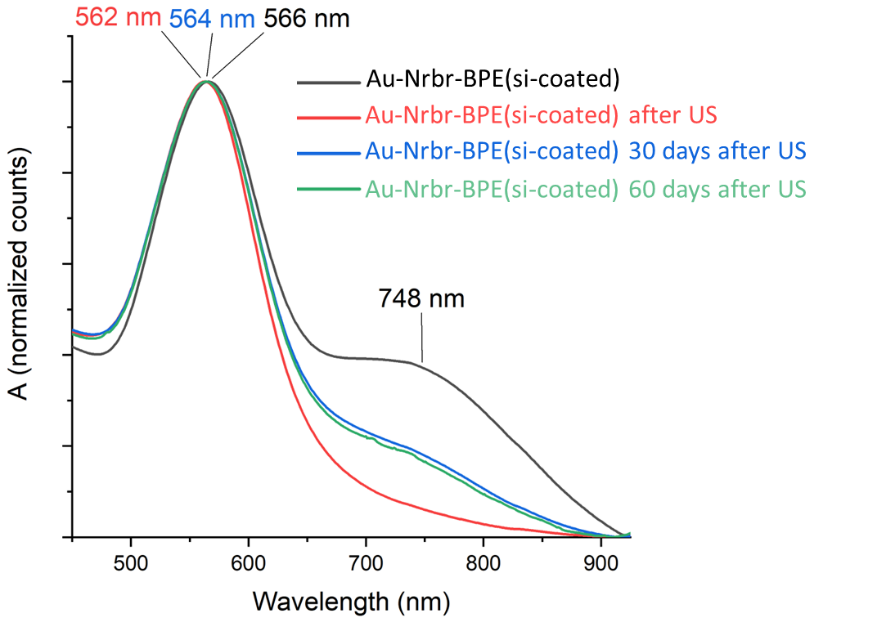


**Fig S2.2** UV-visible absorption spectra of AuNRBs solution before and after the US measurements.

**S3:** **US Power dependence on tissue assembly**

The US power employed in all the standard measurements in the main manuscript was kept at 10 W (20% of the maximum output). This power was intentionally chosen at a level not to incur any detectable thermal damage to the sample during the kinetic measurement. Such thermal damage was observed when the maximum US power was applied (50 W, 100%). Any damage to the tissue sample induced by ultrasound is naturally undesirable and its impact on detected TRS signals of tissue and SERS NPs is illustrated in Figure S2. At this maximum power (50 W), after the measurement, the sample exhibited local discolouration (whitening) consistent with a protein denaturing effect usually seen at raised temperatures (see Fig. S3). This was observed at the point of contact of the US transducer with tissue and at the SERS labelled lesion. Interestingly, this discolouration was not observed between these two affected zones. As the US waves are not focused and radiate into all directions, their power density decreases rapidly with distance from the source indicating that the lesion exhibited an elevated degree of US waves dissipation over the surrounding tissue. This is consistent with an earlier observation that such NPs can exhibit an increased absorption of ultrasound waves and, in fact, are used to increase the effectiveness of some high-intensity ultrasound therapies.^3^ The effect of tissue damage on observed Raman signals also led to the SERS signal intensity decrease. Presumably due to the change of tissue scattering and/or tissue absorption at the tissue zones as a result of the irreversible tissue thermal damage (e.g. lesion) diminishing the overall detectable signals. However, in this instance, also the TRS signal of the tissue matrix decreased making the effect of tissue damage and that of Active SERS easy to differentiate from each other as in appropriate Active SERS experiments with no sample damage where the Raman signal of tissue remained invariant throughout the experiment.


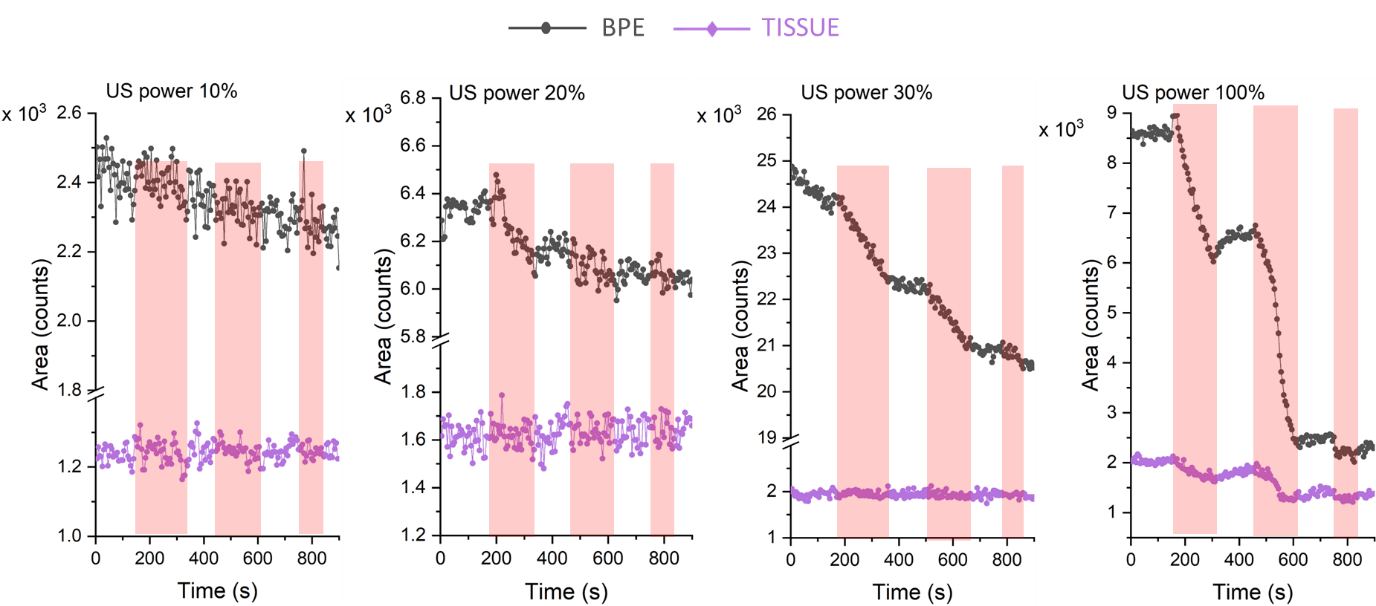


**Figure S3.1**: Raman intensity response to US perturbation by using different powers of the US input (from left to right 10%, 20%, 30% and 100% of 50 W).


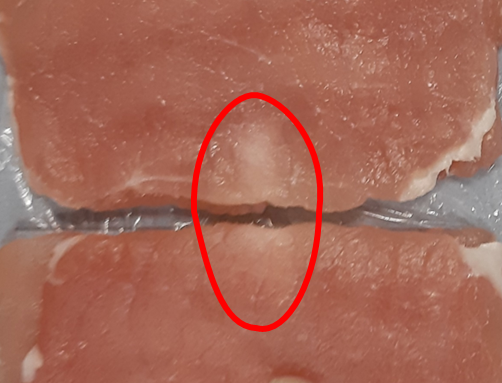


**Figure S3.2:** Photo of the central layer of the sample assembly after the high US power experiment on the inclusion plane (after unfolding the layer). The red oval highlights the ‘whitening’ compatible with the suggestion of thermal damage of the tissue due to the maximum US power of 50 W.

To further prove that the Active SERS signal change in standard measurements is due to the SERS NPs and not the tissue heat effect, we performed an additional measurement, using the same US power as in the main study in this manuscript (i.e. 20% of the maximum power = 10 W), where we embedded PTFE powder uniformly inside the lesion itself along with the NPs. If the tissue damage by US waves were behind the effect of SERS signal intensity decrease observed with 20% US powers, it would also affect to the same degree the spontaneous Raman signals of PTFE located in the same zone as SERS NPs. However, if only SERS signal was selectively responding to US waves, the spontaneous Raman signal of PTFE would remain unchanged throughout the kinetic series. The results of this test are shown in Fig. S4 evidencing that the TRS signal of PTFE signal remained unchanged during the kinetic series in which ultrasound is switched ON and OFF at standard measurement power (10 W). In contrast and as expected, the SERS signal showed a marked decrease in intensity and the tissue signal of the tissue matrix also did not show any intensity changes throughout this series.


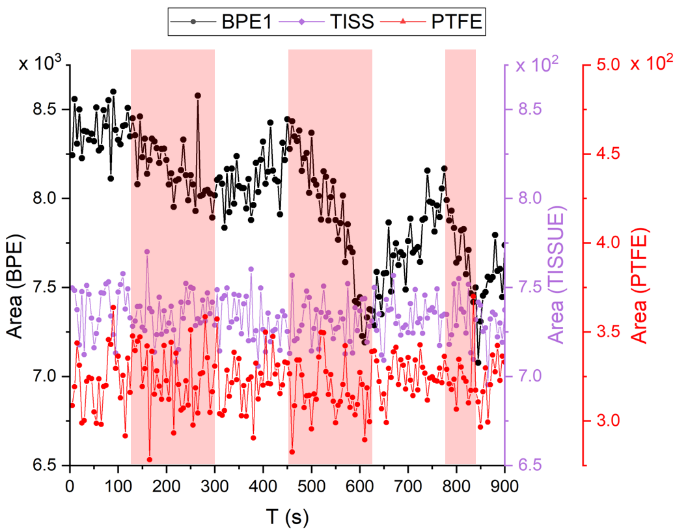


**Figure S3.3:** Raman intensity of BPE (black symbol-line), PTFE (red symbol-line) and tissue (purple symbol-line) response to US perturbation recorded during kinetic TRS measurements with a US power of 10 W.

**S4: Longer term SERS signal intensity recovery after US perturbation**

Longer term SERS intensity recovery phase after the Active SERS measurement was monitored by acquiring a prolonged kinetic acquisition of 600 Raman spectra (1 s x 5 acquisitions each with a total acquisition time of 3000 s = 50 min). During the TRS kinetic acquisition, the external perturbation (US tip) was turned ON only once for 150s (2 min 30 s). The SERS intensity was monitored for the remaining 2700 s after the perturbation. Figure S3.1 shows an overall 77% recovering of the SERS Raman.


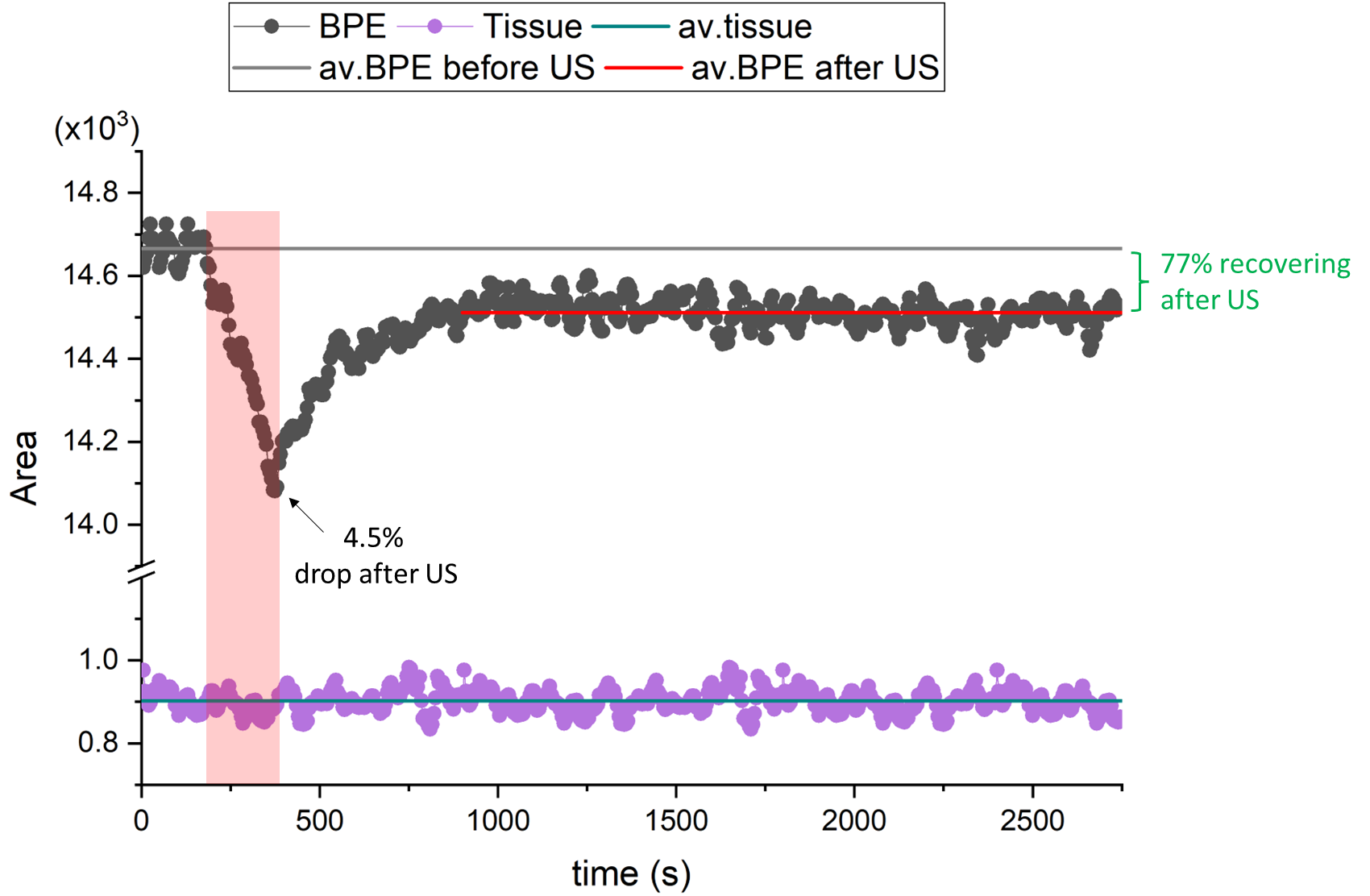


**Figure S4.1:** Raman intensity of BPE (1198 cm^−1^, black symbol-line) and fat (1451 cm^−1^, purple symbol-line) in response to US perturbation. Red shaded area highlights the time interval of the external perturbation. Horizontal line indicates the intensity average before (grey line) and after (red line) the US perturbation.

**References**

1. J. Johnston, E.N. Taylor, R.J. Gilbert, T.J. Webster. “Improved molecular fingerprint analysis employing multi-branched gold nanoparticles in conjunction with surface-enhanced Raman scattering.” Int. J. Nanomedicine. New Zealand, 2016. 11: 45–52. 10.2147/IJN.S93222.

2. K. Chandra, K.S.B. Culver, S.E. Werner, R.C. Lee, T.W. Odom. “Manipulating the Anisotropic Structure of Gold Nanostars using Good’s Buffers”. Chem. Mater. American Chemical Society, 2016. 28(18): 6763–6769. 10.1021/acs.chemmater.6b03242.

3. M. Sadeghi-Goughari, S. Jeon, H.J. Kwon. “Enhancing Thermal Effect of Focused Ultrasound Therapy Using Gold Nanoparticles”. IEEE Trans. Nanobioscience. IEEE, 2019. 18(4): 661–668. 10.1109/TNB.2019.2937327.
